# From disease specific to universal health coverage in Lesotho: successes and challenges encountered in Lesotho’s digital health journey

**DOI:** 10.1093/oodh/oqae021

**Published:** 2024-07-11

**Authors:** Monaheng Maoeng, Kerry Bruce, Maletsatsi Motebang, Carolyn Wetzel Chen, Shirley Lecher, Tsigereda Gadisa, Suzue Saito, Mantebaleng Ntsaba

**Affiliations:** ICT Department, Ministry of Health, Constitution Road, Maseru 100, Lesotho; Department of Compact Operations, Millennium Challenge Corporation, 1099 14^th^ St NW, Suite 700, Washington DC 20005, USA; Centers for Disease Control, Maseru, Lesotho. U.S. Embassy 254 Kingsway Road, Maseru 100, Lesotho; Department of Compact Operations, Millennium Challenge Corporation, 1099 14^th^ St NW, Suite 700, Washington DC 20005, USA; Division of Global HIV and TB, Centers for Disease Control, 1600 Clifton Rd. Atlanta, GA 30333, USA; Strategic Information Department, ICAP at Columbia University, 722 West 168^th^ Street, 13F, New York, NY 10032, USA; Strategic Information Department, ICAP at Columbia University, 722 West 168^th^ Street, 13F, New York, NY 10032, USA; Public Health Department, Millennium Challenge Account, Post Office Building, Kingsway and Constitution Road, Maseru 100, Lesotho

**Keywords:** digital health, electronic medical records, interoperability, Lesotho, governance, security, power, connectivity, human resources

## Abstract

In Lesotho, the Ministry of Health and key donors have made significant advancements to develop digital health solutions specific to HIV services including an eRegister which is interoperable with the health management information system, pharmacy services and the laboratory information system. New investments from the Millennium Challenge Corporation will expand digital health services to all reported communicable and non-communicable disease areas at health facilities throughout the country. This paper explores how digital health interventions designed to support comprehensive HIV care can be leveraged to provide universal digital health coverage. Specifically, three priority areas will be addressed: (i) governance, security, and system architecture (ii) power, connectivity, and equipment (iii) human resources and change management.

## THE DIGITAL HEALTH SYSTEM IN LESOTHO

This rapid communication^1^ describes the implementation of a national electronic medical record and the opportunities and challenges for expansion in Lesotho, a small mountainous country surrounded by South Africa with an estimated population of 2.3 million [1, 2]. The country has the second highest HIV prevalence in the world with an estimated 22.7% of the population 15 years or older living with HIV. As with most high prevalence countries, the burden for women is higher with an estimated 27.4% of women over 15 years old living with HIV, versus men of the same age, an estimated 17.8% [3]. The country has made remarkable strides toward HIV epidemic control. As of 2020, Lesotho attained the Joint United Nations Programme on HIV/AIDS (UNAIDS) 90-90-90 goals [4] and achieved progress toward the 95-95-95 goals [5] which aim to ensure that 95% of all people living with HIV know their status, 95% of all people diagnosed with HIV infection receive sustained antiretroviral therapy and 95% of people receiving antiretroviral therapy have achieved HIV viral suppression. The President’s Emergency Plan for AIDS Relief (PEPFAR), the Global Fund to End HIV, TB and Malaria, UNAIDS and the Government of Lesotho have all made substantial financial, policy and human resource contributions to achieve these goals. The Lesotho government provides universal health coverage (UHC) to its population, although many citizens elect to pay for private health care. A large portion (29%) of health care services in the country is delivered by partners, including the Christian Health Association of Lesotho (CHAL), the Red Cross, and Baylor College of Medicine. The health system is centralized under the Ministry of Health which supports 10 District Health Management Teams (DHMTs) that provide oversight for delivery of health services at 277 public and private health facilities.

Digital health systems have been identified as a priority area for investment in Lesotho to support patient management and program monitoring. The U.S. Centers for Disease Control and Prevention (CDC), with funding from PEPFAR, invested in the development of an electronic medical record (EMR) system for HIV, tuberculosis (TB) and prevention of mother to child transmission (PMTCT) services. This system, called the eRegister, has been implemented as a best practice [5–7] nationwide at 187 of the 277 health facilities, including all hospitals and health centers operated by the Lesotho government, international partners (e.g. CHAL and the Red Cross) and several private facilities. In addition, resources have also been invested in the development of the District Health Information Software 2(DHIS2) system as a sustainable national health information management system (HMIS) for reporting aggregate data for all health programs. Thus, enabling accessibility of facility level data at the national level to inform decisions for program improvement. The DHIS2 is interoperable with the eRegister and the Lab Information System for automated reporting on HIV, TB, and PMTCT related indicators.

The eRegister system was built using the Bahmni project on OpenMRS. *Bahmni* is an open-source electronic medical record and hospital system designed for low resource settings. A technical working group was established, which was composed of government and non-government representatives, to develop terms of reference that led to the development, piloting, and adoption of the eRegister. Three task teams were formed including: (i) The eRegister Advisory Group led by the Lesotho Ministry of Health (MOH), which was responsible for developing policy and strategic direction, while engaging districts and stakeholders. (ii) A Development Core Team which included MOH staff, the International Center for AIDS Care and Treatment Programs, and National University of Lesotho developers that advised on the best distribution of the OpenMRS platform. This group was responsible for the development, piloting, and launch of the eRegister. (iii) The eRegister Task Team composed of MOH technical staff and stakeholders. The Task Team supported the Core Team on the development of business requirements, logic checks, validation and compiling reports. The Development Core Team proposed the use of Bahmni, which combined the OpenMRS with other open-source solutions to meet the MOH information requirements. Bahmni was selected as the best option. Reasons for the selection included the ability to manage complex data, flexibility for expansion to cover multiple diseases, absence of licensing fees and availability of a large community of Africa-based programmers for technical support. Bahmni is also scalable to meet the needs of low- and middle-income countries that must start with limited resources and gradually expand. The MOH, through a strategic information technical working group, updated the HMIS policy (2018) and revised the HMIS strategic plan (2018–24) to provide policy guidance on HMIS advancements, including eRegister development and roll out. The technical working group developed a role-based training package for eRegister users, including health care providers and program managers at the national, district and facility levels.

Success of the eRegister pilot and initial implementation resulted in a phased and decentralized approach to expand the scope and geographic coverage to the national level. The program expanded from a limited focus on anti-retroviral therapy to include comprehensive HIV testing, HIV Care and Treatment, PMTCT services, cervical cancer screening, TB treatment, TB prevention and Pharmacy services. The geographic scope expanded from six health facilities (two hospitals and four health centers) in two districts at the time of the pilot (May–July 2018) to 187 health facilities (17 hospitals and 170 health centers) across the 10 districts of Lesotho, which comprise all of the major health facilities in the country except the national referral hospital. A 29-person implementation team (6 central and 23 district-based) was established to support capacity building and troubleshooting for the system. In 2023, the Laboratory Information System (LIS) integration with the eRegister was developed, piloted, and prepared for national expansion. The LIS integration allowed expanded functionality for ordering and return of essential laboratory tests between the laboratories and clinics.

The Millennium Challenge Corporation (MCC) is returning to Lesotho with a second Compact investment, which began in March 2024 for a 5-year implementation period. In cooperation with the MOH, a major investment in health system strengthening and primary health care is planned. Expansion of the eRegister system will cover all primary health care services in the country by conducting additional assessments of the need [6], and working with stakeholders and the MOH to implement an approach which is aligned with the MOH’s essential health package, updated clinical practice guidelines, and standard operating procedures. The intent of this system is to leverage digital systems to improve reporting, referrals, supply chain management, and overall patient care at clinical facilities throughout the country.

## CHALLENGES FOR THE EXPANSION OF DIGITAL HEALTH SYSTEMS BEYOND HIV AND TB SERVICES

Several challenges remain which need to be addressed to support Lesotho’s expansion of clinical health care services. The three main challenges are described below.

### Governance, security and system architecture

#### Governance and security

The accountability for health information in Lesotho resides with the Government. Partners and donors share in specific responsibilities. The digital health systems have developed at an exponential rate in Lesotho and governance of these systems has not kept pace with required confidentiality policies and privacy standards. The 2019–2023 the eHealth strategy outlined seven priority strategic areas, one of which is Leadership and Governance of Information and Communication Technology (ICT), since implementation has been limited [7].

Digital Health systems in Lesotho are built on open-source platforms which have standardized security features, procedures, and manuals for developers and users. These global resources need to be tailored to the Lesotho setting. A comprehensive threat assessment is planned, and future digital investments need to prioritize management of potential patient data breaches, ransomware attacks, insider threats and social engineering attacks.

There is a need to expand the e-Health Strategy to define priorities for the next 5 years and to secure funding to complete the plan. The MOH needs the assistance of partners to develop policy and standard operating procedures to ensure a well-functioning system. Without ICT governance structures in place, the Ministry will have limited success in managing the IT risks effectively and ensuring activities related to information and technology are aligned with IT governance [8] best practices.

Patient data security is critical for full system functionality. The MOH has adopted user roles for both the eRegister and DHIS2 that control access to confidential information. User passwords are required for all individual users. However, national cybersecurity policies and data encryption processes need to be strengthened and standardized. Support for this work is expected to be included in the forthcoming MCC Compact.

#### System architecture

The MOH plans to work with MCC to define an overall system architecture that is scalable, interoperable, and properly distributed among partners, donors, and government in a collaborative manner to replace disease-specific systems. The objective is to align interventions and to support an interoperable and integral single ecosystem. This structure is intended to coordinate investments, prevent duplication and support balanced participation.

The ‘Lesotho National Digital Transformation Strategy’ [9]outlined a need for interoperability, especially between government systems. While interoperability exists between DHIS2, eRegister, and laboratory information systems for HIV, TB and PMTCT related services, the scope of coordination remains limited. The Ministry of Home Affairs has created a framework for the integration of data through the national identification number, but this has yet to be operationalized in the MOH. There is also a need to plan for a system architecture that is interoperable across diverse government Ministries such as the Ministry of Communications, Science, Technology and Innovation and the Ministry of Finance and Development Planning.

### Power, connectivity and equipment

#### Power

A common problem for all digital health systems in Southern Africa remains reliable and accessible power sources. In Southern Africa, power is a major concern, especially given the recent implementation of a ‘load shedding’ schedule in South Africa [10]. Lesotho bought 40% of its power from South Africa and the health system in some areas of the country experiences frequent power outages. During the MCC Compact I, many of the health facilities that were rebuilt were equipped with solar power systems. However, following 10 years of use, many of these systems are failing because the power they supply did not anticipate the drain of and future needs of the digital health system. Additionally, health facilities relying on solar as their sole power source still experience power outages on cloudy days. Beyond load shedding, health facilities relying on the Lesotho electricity grid experience power outages because they cannot pay for electric credit units, especially during the winter season due to high consumption to heat facilities. Power scarcity was an anticipated challenge that informed eRegister equipment purchases. A decision was made to utilize laptops with battery life long enough to sustain system use for a full working day. Sites were also provided with power banks to keep the mobile wi-fi router charged for 7 days. Despite these low use power solutions and equipment selected to be efficient in low power settings, outages continue to challenge eRegister implementation as many sites go for days without power.

#### Connectivity

Another anticipated crosscutting challenge is the reliability of the internet connection. To overcome this challenge, a decentralized implementation approach was taken despite the eRegister capability to serve as a web-based centralized system. Each site was supplied with a local server (a high-capacity laptop) connected to the workstations using a local area network. Data are captured at points of care across the HIV and TB service cascade including registration, adherence, clinical and pharmacy and synced into the local server without the need for internet connection. Selected patient identification data are extracted to a single, cloud-based platform to allow deduplication and tracking of patient visits across sites. A future system will be more secure and sustainable as it will utilize the Lesotho Government Data Network which utilizes local (not international) bandwidth. Patient data will then be centralized within government data centers resulting in improved security and lower risk of a data breach through the internet.

#### Equipment

Throughout the implementation, providers have generally shown a preference for laptops over tablets. The need for long battery life laptops over desktops has been critical to overcome the problem of frequent power outages. Screen touch laptops at the early stages of implementation helped to build on users’ mobile phone experience and shorten the learning curve. However, as implementation matured, users have started using keyboards, lessening the importance of screen touch laptops.

An additional challenge is how to ensure the MOH allocates a sufficient budget for replacement equipment on a routine basis. To date, the MOH has relied on donors for the procurement of equipment, rather than allotting this cost into their recurring budget. If the digital health investments are to be sustained, a method to ensure recurring costs are recognized and planned for will be essential.

### Change management and human resources

The people who implement the system are at the heart of its potential success or failure. Other challenges that have been identified include: the number of people available, their skill level and their willingness to implement the digital health program.

#### Human resources for ICT

The MOH ICT Unit is acutely understaffed. Implementing partners have contributed staff to support the unit, but the longevity of these positions is not guaranteed. The MOH is considering restructuring. There is an opportunity to rethink the number and type of staff who support digital health at the central ministry and at the district level. MCC will also support the Ministry of Information, Communications, Science, Technology, and Innovation to stand up a service contract to provide technical support at the district level, initially for digital health, but eventually for a wider array of digital investments.

At the facility level, implementing partners have provided clerks to support digital health systems, specific to HIV and TB data. But if the future system will encompass all reportable diseases, a re-organization of staffing support must be considered to centralize registration and data analysis.

#### Technical capacity

The vast majority of deep technical capacity to design, stand up and implement digital health systems has been provided by partners. A positive impact of the financial support and training has been building human capacity for Lesotho, as most of the trained staff are Basotho (indigenous people native to Lesotho). MOH officials need to be fully informed and engaged with what is being implemented to ensure ownership of the expanding digital health system.

#### Change management

Health care workers have at times expressed concern that digital health systems would bring additional work. The eRegister was designed to simplify their work by removing redundant recording tasks and transform an inefficient manual reporting system to a seamless automated process where eRegister data is aggregated into the DHIS2. The eRegister’s interface and its ability to instantly convert patient data into useful visuals have supported point of care decision making (e.g. patient dashboard, viral load reminders, prompts and alerts). The eRegister also supports logic checks to guide compliance with clinical guidelines necessary to provide state of the art clinical care. Individualized, role-tailored, hands-on mentorship for clinical users has also aided system adoption. However, some duplicate paper-based systems remain, and the benefits of a purely digital system have not been fully realized.

## CONCLUSION

The Digital Health System in Lesotho and specifically the eRegister is well positioned to scale from a single disease area to encompass the entire primary health care system and provide more equitable care to the population. However, formidable challenges remain. A system that was built to serve only HIV and TB needs to be expanded in multiple areas to become functional across all reportable diseases. Comprehensive assessments and pilots, prior to major investments will be key to inform system scope, procurements and planning for implementation.

Open-source technologies, when carefully architected, can provide building blocks for expanded systems if core elements of digital health systems including governance, security, power, and connectivity are in place. A comprehensive approach, addressing the components, systems and the people involved will be the best approach to ensure a system will work as intended.

Expanding the digital health system to all reported disease area stands to improve the quality of Lesotho’s UHC system. A universal digital health system will improve the continuity of care for the entire population of Lesotho, not just those with HIV and TB, and improve the availability of data and evidence for decision making.

## Data Availability

No new data were generated or analyzed in support of this article.

## Footnotes

1 Please see the disclaimer in the Study Funding section.
