# Correction to: From disease specific to universal health coverage in Lesotho: successes and challenges encountered in Lesotho's digital health journey

**DOI:** 10.1093/oodh/oqae038

**Published:** 2024-09-12

This is a correction to: Monaheng Maoeng, Kerry Bruce, Maletsatsi Motebang, Carolyn Wetzel Chen, Shirley Lecher, Tsigereda Gadisa, Suzue Saito, Mantebaleng Ntsaba, From disease specific to universal health coverage in Lesotho: successes and challenges encountered in Lesotho's digital health journey, *Oxford Open Digital Health*, Volume 2, 2024, oqae021, https://doi.org/10.1093/oodh/oqae021

In the originally published version of this manuscript, in the section entitled THE DIGITAL HEALTH SYSTEM IN LESOTHO, the estimated population of Lesotho was incorrectly given as 23 million, instead of 2.3 million.

Additionally, (CHAL) was incorrectly stated to stand for the Christian Health Alliance of Lesotho, instead of the Christian Health Association of Lesotho.

These errors have been corrected.

